# Morbidity and mortality in adults with congenital heart defects in the third and fourth life decade

**DOI:** 10.1007/s00392-022-01989-1

**Published:** 2022-03-01

**Authors:** Matthias J. Müller, Kambiz Norozi, Jonas Caroline, Nicole Sedlak, Jonas Bock, Thomas Paul, Siegfried Geyer, Claudia Dellas

**Affiliations:** 1 Department of Pediatric Cardiology and Intensive Care Medicine, Georg August University, Robert-Koch-Str. 40, 37075 Goettingen, Germany; 2 Pediatric Cardiology, Western University, London, ON Canada; 3 Medical Sociology Unit, Hannover Medical School, Hannover, Germany

**Keywords:** Adults with congenital heart defect, Morbidity, Mortality, Congenital heart defect, Observational study

## Abstract

**Objectives:**

The population of adults with congenital heart defects (ACHD) is continuously growing. Data on morbidity and mortality of ACHD are limited. This longitudinal observational study examined a group of ACHD with surgically corrected or palliated congenital heart defects (CHD) during a 15-year period.

**Methods:**

ACHD that had participated in the initial study were invited for a follow-up examination. Mortality and hospitalization data were compared with a healthy control group.

**Results:**

From 05/2017 to 04/2019 a total of 249/364 (68%) ACHD participated in the follow-up study: 21% had mild, 60% moderate and 19% severe CHD. During the observational period, 290 health incidents occurred (cardiac catheterization 37%, cardiovascular surgery 27%, electrophysiological study/ablation 20%, catheter interventional treatment 14%, non-cardiac surgery 3%). Events were more frequent in ACHD with moderate (53%) and severe (87%) compared to those with mild CHD (*p* < 0.001). 24 individuals died at a median age of 43 years during the observation period. 29% of them had moderate and 71% severe CHD corresponding to a mortality rate of 0%, 0.29% and 1.68% per patient-year in ACHD with mild, moderate and severe CHD. Long-term survival was significantly reduced in patients with severe CHD in comparison to individuals with mild and moderate CHD (*p* < 0.001).

**Conclusion:**

After correction or palliation of CHD, there was remarkable ongoing morbidity and mortality in ACHD patients over the 15-year observation period, particularly in individuals with moderate and severe CHD when compared with the general population. Thus, life-long special care is required for all surgically corrected or palliated ACHD patients.

**Graphical abstract:**

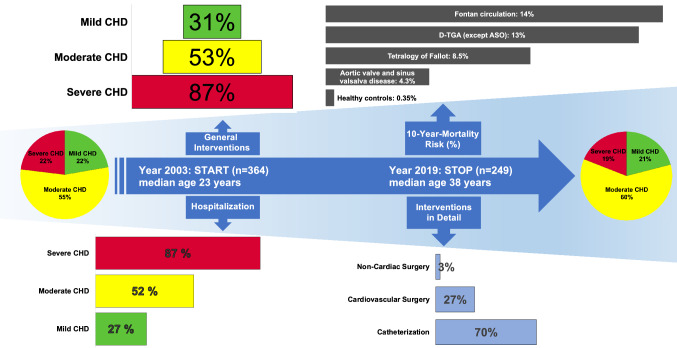

## Introduction

About 1% of all live births suffer from a malformation of the heart or great vessels. Thanks to advances in diagnostics and treatment, more than 90% of children with the whole spectrum of congenital heart defects (CHD) are reaching adulthood now. Thus, the population of adults with congenital heart defects (ACHD) is continuously increasing and aging [1]. In general, ACHD represents a relatively new patient population with a broad variety of CHD from mild to severe [2]. Regardless of severity, almost all patients with CHD suffer from chronic heart disease, which requires regular follow-up care by CHD-specialized cardiologists. Particular attention must be paid to potential residuals of corrected or palliated CHD and additional comorbidities [3]. The present longitudinal observational study was designed to examine the long-term course of patients with surgically corrected or palliated heart defects over a period of 15 years in a tertiary ACHD facility by examining the morbidity and mortality in comparison with controls from the general population.

## Patients and methods

**Fig. 1:**
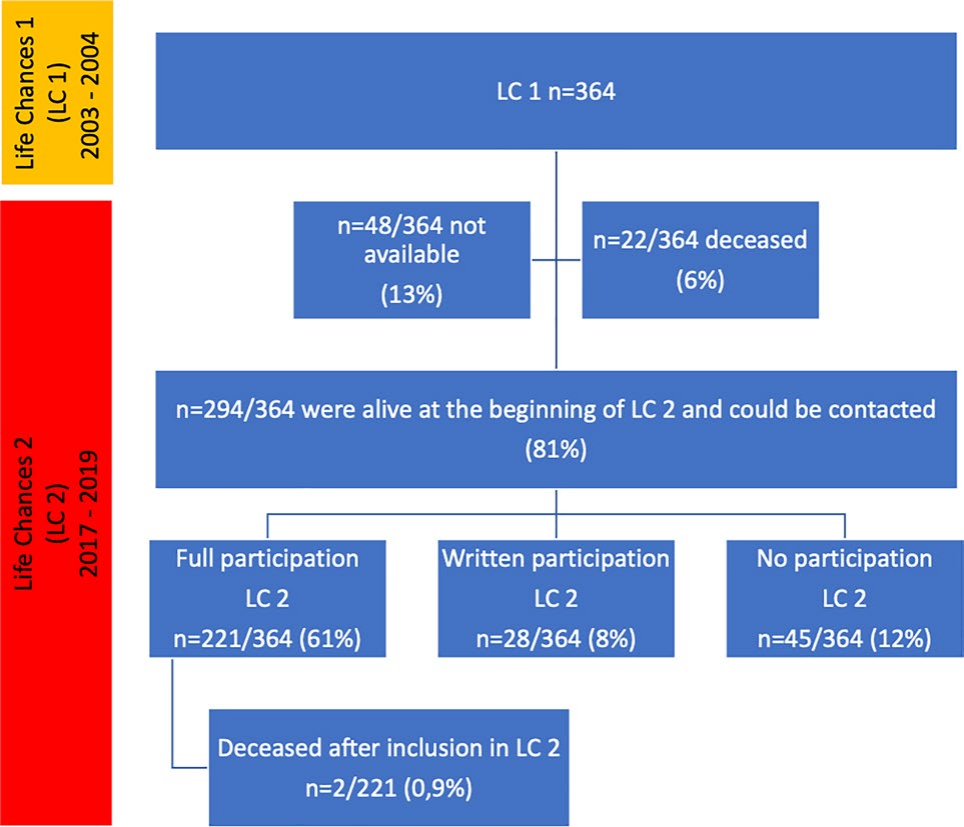
Flowchart of patients who participated in LC2 (LC: Life Chances)

In a previous cross-sectional study in our center from 2003 to 2004 (reviewed and approved by the ethics committee of Hannover Medical School under no. 3710, date: 04-10-2004 and by the University Clinic of Goettingen under no. 10/2/01, date: 01-03-2001) entitled *Life Chances 1* (LC1), a total of 364 patients with various types of corrected or palliated CHD had been extensively studied [4, 5]. These patients had a median age of 24 (range 14 to 45) years. For the current study, *Life Chances 2* (LC2), all 364 patients from LC1 were contacted by phone, via mail, or via their general practitioners (Fig. 1). Between 05/2017 and 04/2019, all patients were invited to our outpatient clinic for follow-up examination that included medical history, physical examination, ECG, 2D- and 3D-echocardiogram, blood sampling, exercise stress testing, and socio-medical interview (reviewed and approved by the ethics committee of the University of Goettingen, no. 15/8/14). Complexity of CHD was defined as mild, moderate, and severe according to the 2020 ESC Guidelines for the management of adult congenital heart disease [1]. In patients with multiple cardiac lesions, the lesion of highest complexity was assigned.

To evaluate the findings on morbidity and mortality in the patient sample, control groups were drawn from the German Socio-Economic Panel (SOEP) as a national longitudinal German population survey. Controls for comparing morbidity were drawn from the survey 2004 by using age, gender, and parental education as matching variables. Parental education was used as a replacement for own education as many patients had not completed school education when the first survey was conducted. Finally, 363 cases with longitudinal records were drawn. For mortality, 1089 controls were available as also shorter observation periods could be allowed for.

### Patient and public involvement

Patients were not involved in planning and realization of this project. During the initial cross-sectional study from 2003 to 2004, patients with various types of corrected or palliated CHD were consecutively recruited during visits from the outpatient clinic of the Department of Pediatric Cardiology and Intensive Care Medicine, Georg August University Medical Center, Goettingen, Germany. Between 05/2017 and 04/2019, all patients were invited to the outpatient clinic for follow-up examinations. After the first and the second study, findings were presented and discussed in a symposium for patients.

### Statistics

Statistical analyses were performed using SPSS® 26.0 (IBM, New York, USA). Numerical data are presented as median and interquartile range (IQR). Differences between numerical data were calculated using non-parametric tests, Mann–Whitney-*U* test, or Kruskal–Wallis test. Differences between variables were calculated by Chi-square test. Patient-years (py) were calculated as total years between inclusion into LC1 and LC2 or, if deceased, until date of death. For patients with an unknown date of death, 05/31/2017 was inferred as the date of death which was the start of *LC2*. Mortality was calculated by dividing the number of deaths by total patient-years between LC1 and LC2. Long-term survival is displayed by Kaplan–Meier survival curves and was tested for significance by the log-rank test. Risk for death was calculated by Cox regression analysis and described as hazard ratio (HR). A *p* value < 0.05 was defined as error level.

## Results

### Study population

Of the 364 individuals of LC1, a total of 221 patients (61%) followed the invitation for the follow-up examination for LC2 (Fig. 1). Another 28/364 (8%) patients completed a socio-medical questionnaire only, resulting in a total attendance of 249/364 patients (68%) at a median age of 38 (IQR: 33–47) years, while the youngest patient was 27 and the oldest was 60 years of age, respectively.

The remaining 115/364 (32%) patients did not participate in LC2. The reasons included:

1. The patient could not be reached/was lost to follow-up (48/364; 13%).
2. 45/364 (12%) patients refused to participate in LC2. Of those, 20% had a simple, 47% a moderate and 33% a severe CHD, respectively. 14/45 (31%) patients had been studied in LC1 and had not further regular cardiac checkups thereafter. The remaining 69% (31/45) attended regular cardiac checkup at our tertiary ACHD facility, but could not be motivated to participate in LC2.
3. The patient had died (22/364; 6%). Two others died shortly after inclusion in LC2, totaling 24 deaths. All patients who could be contacted but refused to participate in LC2 were assigned the status "alive" at the time of LC2 (Fig. 1).

### Follow-up visits from LC1 to LC2

Two patient questionnaire surveys were conducted. Initially, patients were asked during data collection for LC1 and subsequently during data collection for LC2.

For clinical examination, there were no exactly defined periods of follow-up visits between LC1 and LC2. 63% of LC2 study patients had presented to regular cardiology examinations, i.e., at least once every 5 years. The remaining 37% had not undergone regular cardiac follow-up assessment (*p* < 0.05). The severity of CHD had an impact on regular cardiologic follow-up visits: 35% with mild CHD had regular follow-up visits vs. 64% with moderate and 89% with severe CHD.

### Cardiac malformations

**Fig. 2:**
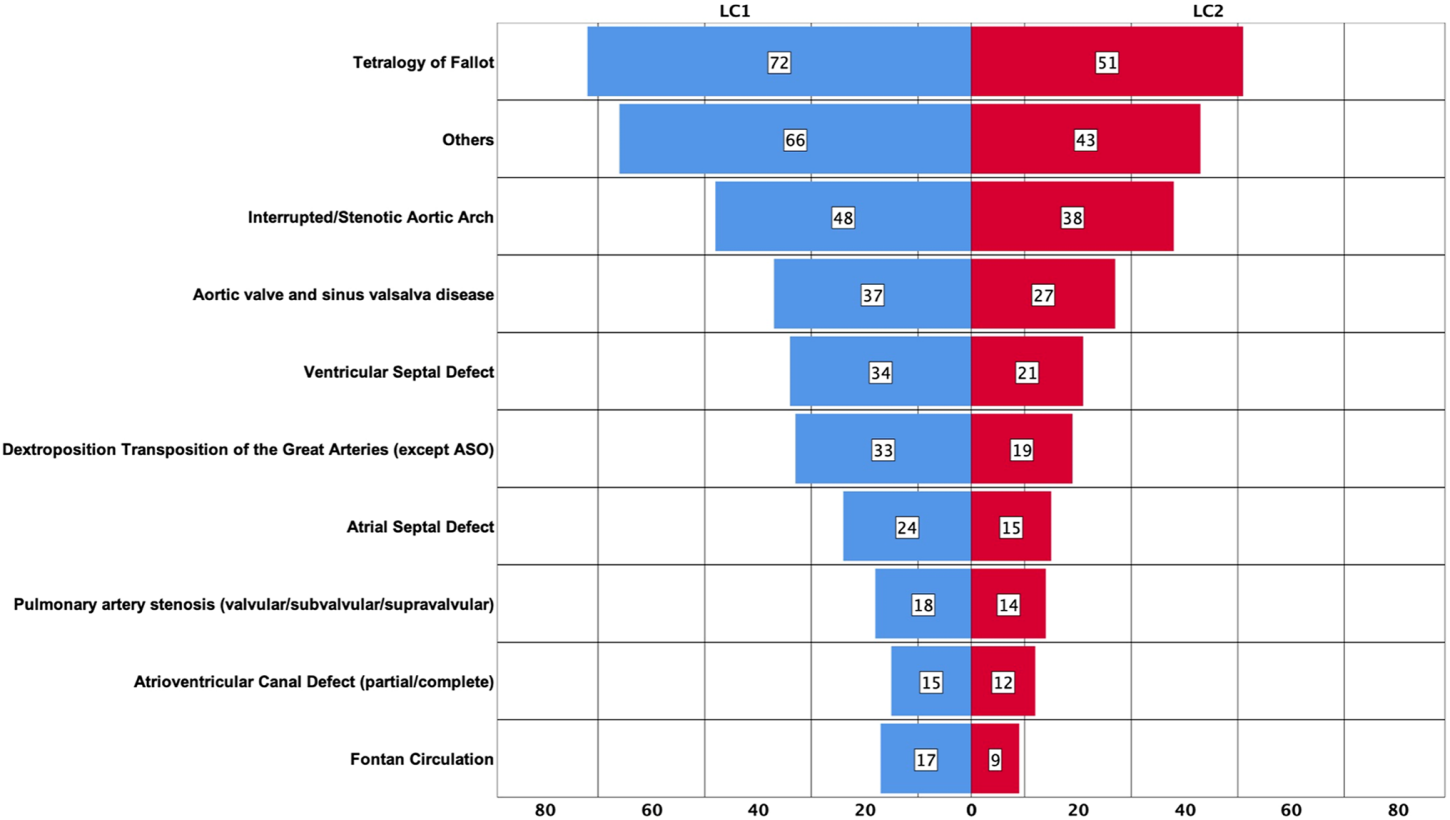
Vertical bar charts show the number of patients per diagnosis group at LC1 (left bar chart) and, at LC2 (right bar chart)

For detailed analyses of LC1 and LC2, all cardiac malformations present in more than 14 individuals in LC1 were grouped as a specific entity. Rare cardiac defects involving less than 14 patients were classified as "others". All patients with univentricular hearts who had any type of a Fontan Circulation were assigned to “Fontan Circulation” irrespective of the specific underlying cardiac malformation. In this way, all patients could be assigned to ten different diagnosis groups (Fig. 2). Detailed basic information from all patients of LC1 and LC2 are displayed in Table 1.

**Table 1:** Detailed patient characteristics according to the ten diagnosis groups at LC1 and LC2

	Congenital heart defect (*n*; %)	Gender Female (*n*; %)	Age in years (median; range)	*p* value	Body mass index (median; range)	*p* value
	*Aortic valve and sinus valsalva disease*					
LC I	37/364; 10%	8 (22%)	27.0; 14.0–43.0		23.7; 16.9–38.0	
LC II	27/249; 11%	4 (15%)	41.0; 28.0–58.0	p < 0.001	25.2; 19.1–49.6	*p* = 0.01
	*ASD*					
LC I	24/364; 7%	14 (58%)	19.5; 14.0–42.0		23.1; 16.0–37.6	
LC II	15/249; 6%	9 (60%)	35.0; 27.0–56.0	p < 0.001	23.7; 19.0–33.5	*p* < 0.023
	*Atrioventricular canal defect (partial/complete)*					
LC I	15/364; 4%	8 (53%)	23.0; 15.0–39.0		22.1; 17.4–34.2	
LC II	12/249; 5%	7 (58%)	39.5; 29.0–52.0	*p* < 0.001	24.1; 19.9–36.1	*p* = 0.003
	*Dextroposition transposition of the great arteries except atrial switch operation*					
LC I	33/364; 9%	7 (21%)	22.0; 16.0–42.0		22.2; 16.6–34.8	
LC II	19/249; 8%	4 (21%)	35.0; 30.0–49.0	*p* < 0.001	25.7; 18.1–33.6	*p* = 0.002
	*Fontan Circulation*					
LC I	17/364; 5%	9 (53%)	29.0; 14.0–39.0		22.1; 17.6–34.2	
LC II	9/249;4%	5 (56%)	37.0; 29.0–52.0	*p* < 0.001	22.4; 20.8–33.3	*p* = 0.008
	*Interrupted/stenotic aortic arch*					
LC I	48/364; 13%	18 (38%)	23.0; 14.0–42.0		23.7; 16.3–32.4	
LC II	38/249; 15%	16 (42%)	38.0; 28.0–56.0	*p* < 0.001	25.6; 17.0–33.7	*p* = < 0.001
	*Pulomonary artery stenosis (valvular/subvalvular/supravalvular)*					
LC I	18/364; 5%	8 (44%)	23.5; 14.0–44.0		23.6; 17.5–33.5	
LC II	14/249; 6%	6 (43%)	37.0 28.0–58.0	*p* < 0.001	26.3; 17.0–45.4	*p* = 0.027
	*Tetralogy of Fallot*					
LC I	72/364; 20%	34 (47%)	32.0; 15.0–44.0		23.6; 16.9–31.5	
LC II	51/249; 20%	22 (43%)	47.0; 29.0–59.0	*p* < 0.001	24.9; 20.7–41.0	*p* = < 0.001
	*Ventricular septal defect*					
LC 1	34/364; 9%	13 (38%)	21.0; 14.0–45.0		23.5; 18.9–34.7	
LC II	21/249; 8%	9 (43%)	36.0; 28.0–60.0	*p* < 0.001	25.7; 19.0–45.0	*p* = 0.016
	*Others*					
LC I	66/364; 18%	35 (53%)	23.5; 14.0–44.0		22.7; 16.3–35.3	
LC II	43/249; 17%	23 (54%)	35.0; 27.0–58.0	*p* < 0.001	25.1 16.0–39.7	*p* = 0.002

### Severity of CHD

Distribution of CHD severity among participants of LC1 and LC2 did not differ significantly. Severity of CHD of the 364 patients (female *n* = 154, 42%) of LC1 was as follows: mild in 81 patients (22%), moderate in 199 (55%) and severe in 84 (23%). Of the 249 patients in LC2 (female *n* = 105, 42%), 52 patients (21%) had mild, 150 (60%) had moderate and 47 (19%) had severe CHD (n.s.).

In contrast, distribution of CHD severity was significantly different in patients who were lost to follow-up (*n* = 48) compared to patients of LC2. Patients who were lost to follow-up had significantly more often mild than moderate and severe CHD, respectively (*p* = 0.001). In general, the inclusion rate between LC1 and LC2 decreased for each CHD group by approximately 20–30%. It is of note, that the highest non-responder rates were present in the groups of patients with dextroposition transposition of the great arteries (dTGA, 14/33; 42%) and Fontan Circulation (8/17; 47%). 5/14 (36%) patients with dTGA had died between LC1 and LC2, 3/14 (21%) could not be reached and 6/14 (43%) did not want to participate in LC2. Reasons for non-participation in LC2 for patients with a Fontan circulation were death in 3/8 (38%), lost to follow-up in 1/8 (13%) or refusal to participate in 4/8 (50%).

### Hospitalizations

**Fig. 3:**
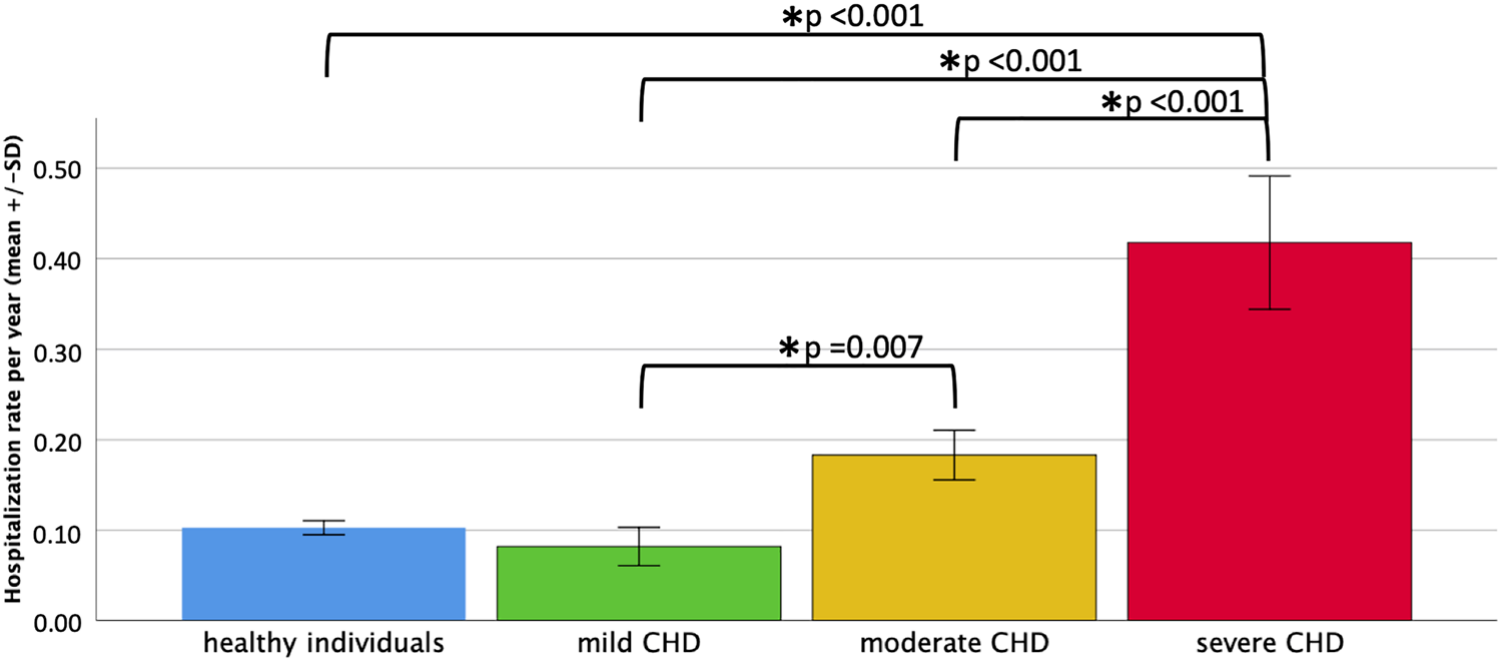
Hospitalizations per year (mean ± SD) of patients with mild, moderate and severe CHD and healthy individuals

Hospitalization records were available from 234 individuals who participated in LC2. Of those, 147 (63%) needed hospitalization during follow-up. 127/147 (86%) ACHD patients were admitted to the hospital for cardiac reasons. Annual hospitalization rates increased with severity of CHD (Fig. 3). Compared to 238 healthy controls matched for age, sex and education, hospitalization rates were significantly higher for patients with CHD than for controls (p = 0.008). However, this finding was mainly related to patients with severe CHD (Fig. 3).

### Morbidity

A total of 290 interventions were performed between *LC1* and *LC2* including cardiac catheterization (*n* = 106/290; 37%), cardiovascular surgery (*n* = 79/290; 27%), electrophysiological study (EPS) and/or catheter ablation (*n* = 57/290; 20%), interventional catheterization (*n* = 39/290; 13%) and non-cardiac surgery (*n* = 9/290; 3%). Interventions were more frequent in patients with moderate CHD (78/146 patients, 53%) and severe CHD (41/47 patients, 87%) than in individuals with mild CHD (15/48 patients, 31%; *p* < 0.001). Importantly, this effect was not influenced by age, as there was no age difference between individuals with mild (median age 36.0; IQR 33.0–43.3 years), moderate (median age 40.0; IQR 33.8–48.0) and severe CHD (median age 35.0; IQR 33.0–43.0 years; *p* = n.s. for each group tested against each other).

### Mortality

**Fig. 4:**
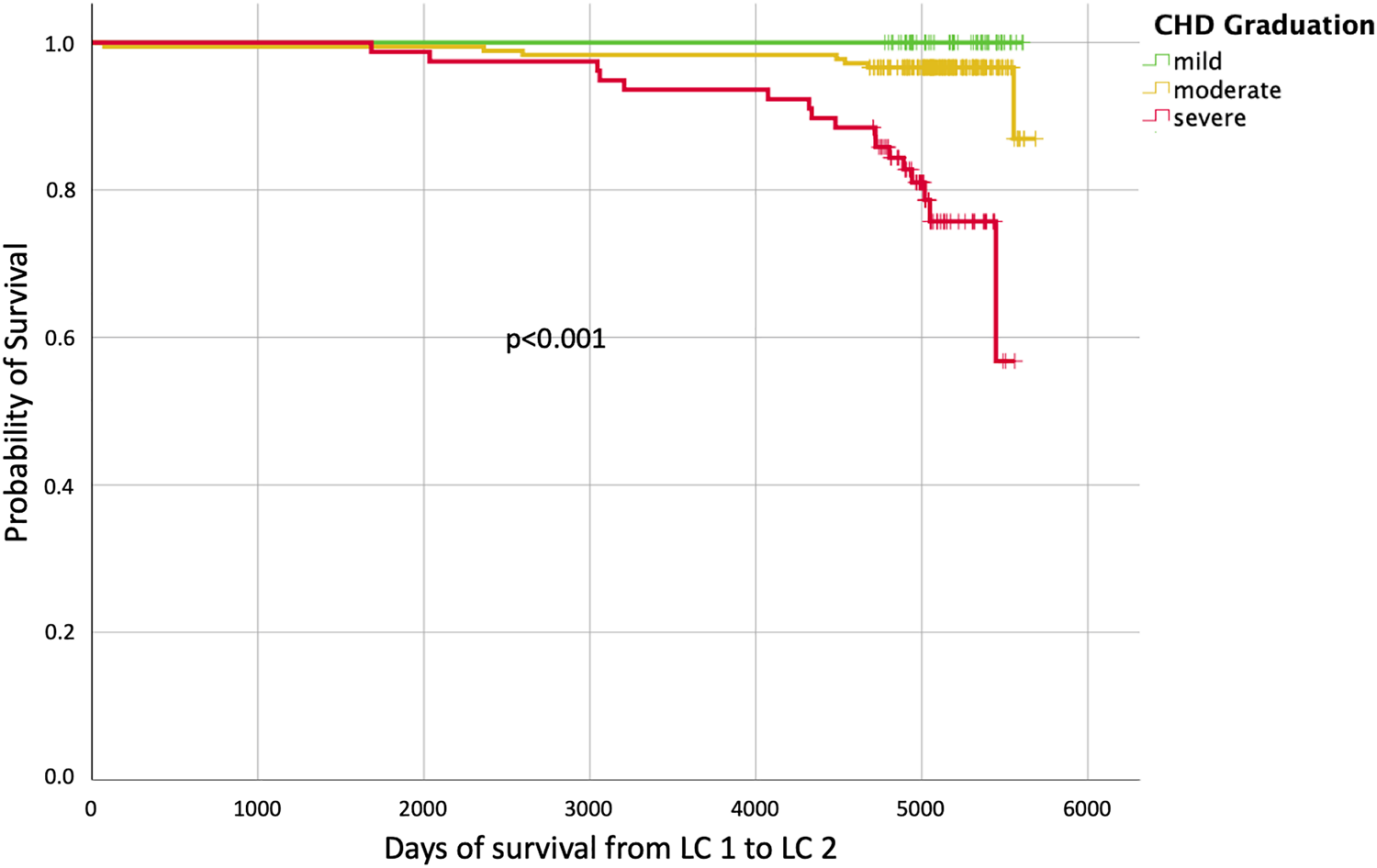
Survival from LC1 to LC2

**Fig. 5:**
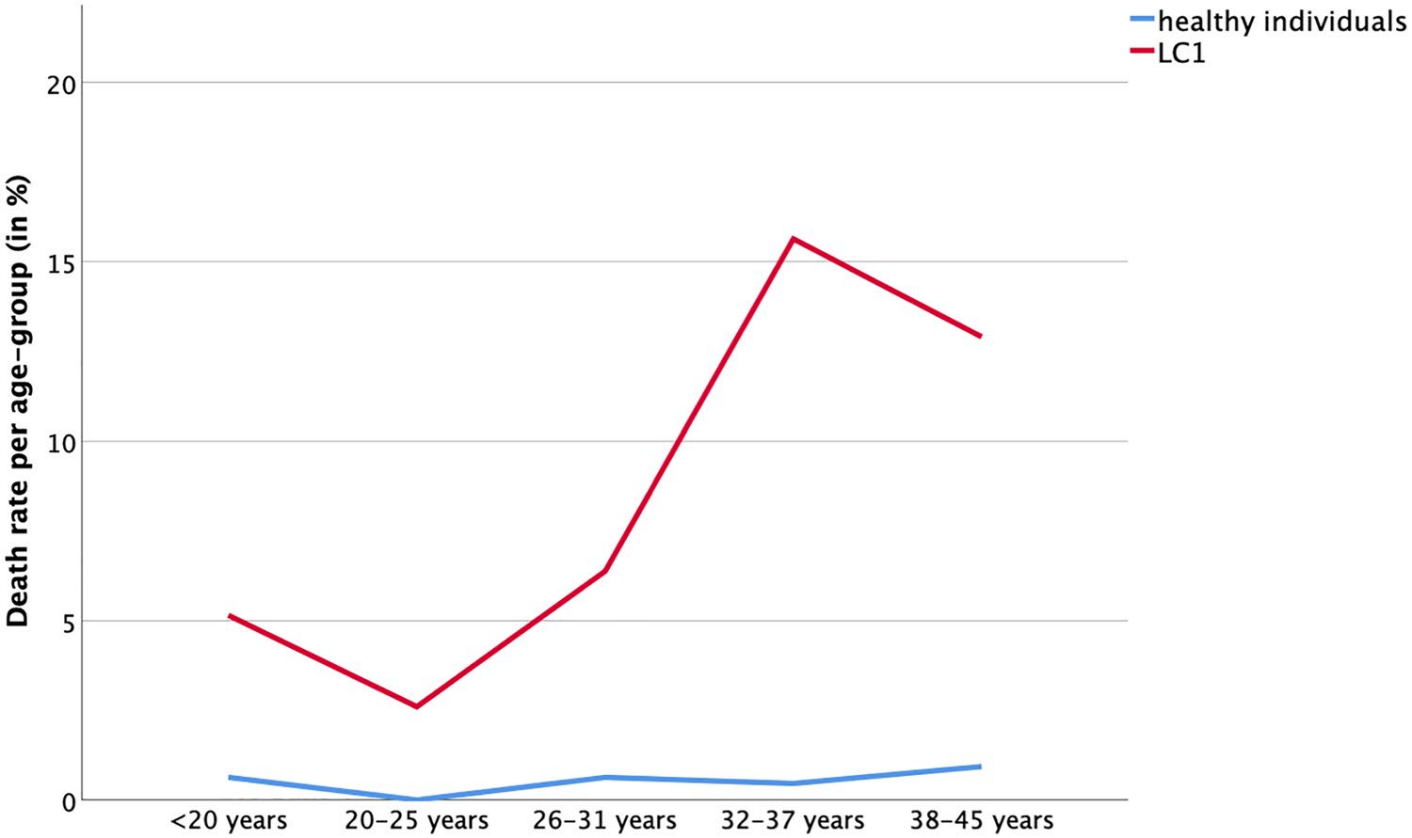
Death in controls (blue line) to study population with CHD (red line). Age at LC1 was used as the baseline for statistical analysis. There was a significant difference in death between controls and CHD patients in each age group (*p* ≤ 0.012)

For assessment of mortality, all patients who were not available at LC2 (*n* = 48) were excluded from further analysis. Thus, survival status at LC2 was known from 316/364 (87%) patients. During the follow-up period between LC1 and LC2 covering a total of 4.285 patient-years, 24/316 patients had died (8%) yielding a total mortality rate of 0.56%/patient-year. In patients with severe CHD, long-time survival was significantly impaired when compared to patients with mild and moderate CHD (*p* < 0.001; Fig. 4). Patients had died at a median age of 43 (IQR: 38–49) years. Causes of death were known in 14/24 (58%) patients, of which 71% were related to CHD (Table 2). None of the deceased patients had mild CHD, while 7 (29%) had moderate and 17 patients (71%) had severe CHD. Women and men were equally distributed among the deceased individuals. Figures revealed a mortality rate of 0% in the mild, 0.29% in the moderate, and 1.68% in the severe CHD group per patient-year, respectively. When compared to patients with mild and moderate CHD, patients with severe CHD had a nearly eightfold higher risk of death (HR 7.97, IQR 1.12–56.62, *p* = 0.038) than patients with mild CHD and a sixfold higher risk of decease (HR 6.25, IQR 2.58 to 15.13, *p* < 0.001) than individuals with moderate CHD, respectively. The highest mortality rate occured in patients with Fontan Circulation (19%, 1.4%/patient-year) followed by patients with dTGA after atrial switch operation (16%, 1.3%/patient-year; see Table 3). When compared to healthy controls matched for age, sex, and education, patients with CHD had a 16-fold increase in mortality during the study period. During the same time, 5/1089 (0.46%) healthy individuals had died reflecting a mortality of 0.035% per person-year (*p* < 0.0001). In healthy controls, mortality ranged between 0 and 1% per 5-year-age group for all ages including those individuals who had already been in the “higher” age groups above 31 years at LC1 (Fig. 5). In contrast, patients with CHD had a significantly increased mortality compared to their age-matched healty controls for all age groups (*p* < 0.05 per age group). This finding emerged irrespective of younger or older age at the beginning of the study, revealing the greatest difference for those individuals above 31 years (Fig. 5).

**Table 2:** Details of the congenital heart defect, the cause of death (if known) and the patient's age at the time of death

No	CHD	Sex	Causes of death	Age at death (year)
1	AS	M	Unknown	36
2	AS	M	Unknown	45
3	ccTGA	M	< 90 days after heart transplantation with complicated course	46
4	ccTGA	M	Unknown	Unknown
5	DILV, Fontan	F	Failing Fontan	28
6	DORV	F	Unknown	Unknown
7	DORV	M	Unknown	Unknown
8	dTGA, Mustard	M	Liver carcinoma	51
9	dTGA, Mustard	M	Unknown	Unknown
10	dTGA, Mustard	F	Unknown	28
11	dTGA, Mustard	M	Unknown	Unknown
12	dTGA, Rastelli	M	Decompensated heart failure	47
13	HCM	M	Decompensated heart failure	39
14	M. Ebstein, Fontan	F	Pulmonary aspergillosis due to immunosuppression after heart transplantation 14 months ago	48
15	Morbus Ebstein	F	Decompensated heart failure with multiorgan failure	40
16	PA, Fontan	M	VT/VF	39
17	PA, Fontan	F	Renal failure	42
18	PA, Fontan	F	unknown	unknown
19	PA, Fontan	M	unknown	39
20	TA, Fontan	M	Failing Fontan and heart transplantation 2 days ago	44
21	TA, Hemifontan	F	< 90 days after heart transplantation with complicated course	54
22	TOF	F	Endocarditis	53
23	TOF	f	Metastatic colon carcinoma	50
24	TOF	f	Aspiration	31

**Table 3:** Annual mortality risk (%) according to the diagnosis groups

Patient group with cases of death	Mortality risk per patient-year (%)
Aortic valve and sinus Valsalva disease	0.43
Tetralogy of Fallot	0.85
Others	0.87
Dextroposition transposition of the great arteries except atrial switch operation	1.3
Fontan circulation	1.4
Total	0.56
Healthy individuals	0.035

### Socio-medical interview

*Data on the socio-medical interview were collected and analyzed by the Medical Sociology Unit, Hannover Medical School. A focus was placed on "Life chances after surgery of congenital heart disease". The educational and occupational performance of patients over the 15 years period was examined. Patient data were compared with a control group generated from the German Socio-Economic Panel (SOEP), which included subjects from the first survey (2003–2004) and who were already included in LC2. Our patients, when compared with the control group, did not exibit any differences in intergenerational educational mobility. When compared for the intragenerational social mobility, however, there were more frequent downward changes* [6].

## Discussion

This longitudinal observational study provides long-term data of an ACHD population—in the middle of their life from a single tertiary ACHD center revealing high morbidity and mortality compared to age-matched healthy controls. Of particular importance is the fact, that patients studied were not limited to a singular entitiy of CHD, but represented the entire spectrum from mild to severe CHD.

Due to advances in diagnostics, interventions, surgery, and care for ACHD in the last decades, the number of ACHD reaching adulthood has steadily increased [7]. In childhood survivors of CHD, the mortality rate has declined by a comparable amount to the general public [8]. These fortunate findings are particularly applicable in patients with mild CHD who have shown comparable [9] or only slightly elevated mortality rates [10, 11] when compared to the general population as found in our study. None of the patients with mild CHD had died during the study period, while we were able to focus on a significantly longer follow-up period than previous studied [2]. It is of note, however, that we included fewer patients with mild CHD (21%) compared to previous reports and to what is expected in the overall ACHD population (55%) [7]. Our data reflect the pattern of care of ACHD patients in a large tertiary ACHD facility covering more patients with moderate and severe cardiac malformations. As reported before [9, 12], our patients with moderate and particularly severe CHD exhibited an increased mortality during the follow-up period. This finding is significant as our patients had already been “long-term survivors”, i.e., survivors of childhood, when they had entered into LC1. Taking the potential study population at LC1 as a point of departure [13], 5% had died, and another 16% did not respond. Considering all patients of this study, mortality correlated strongly with severity of underlying CHD. Altogether, ACHD patients of the present study had a 16-fold higher mortality rate than expected for age-matched healthy controls. Moreover, patients with severe CHD had a significantly increased risk of death than patients with mild and moderate CHD.

Currently, long-term survival for patients with univentricular circulation palliated by the contemporary modifications of the Fontan procedure improved to a predicted 30-year survival of approximately 85% and without a sudden decline in survival or increase in mortality so far [14]. Almost 90% (15/17) of our patients with a Fontan Circulation had been palliated before 1995, while the earliest procedure had been performed in December 1970. These patients had a median age of 29 years at LC1 and developed a remarkably high mortality rate of 14% per 10 years. Almost equally, a high mortality rate was also obserevd in patients with dTGA after atrial switch procedures. It is known that childhood survivors after atrial switch operation have a relatively stable period in their second and third decade of life with accelerated morbidity and mortality rate thereafter [15]. For example, in 91 consecutive patients who had a Mustard repair before 1980, Cuypers et al. calculated a cumulative survival of 84% after 10 years, 80% after 20 years, 77% after 30 years, and 68% after 39 years, respectively [16]. Data reflect a mortality rate of 0.3% per year for patients in their 20s and 1% per year in their 30s. At the start of LC1, nearly 90% of our dTGA patients were < 30 years of age and only one had been operated after 1988. Yet, in their 20s, these patients had a high mortality rate (13%) during the following decade. Slightly lower than patients with Fontan Circulation or dTGA after atrial switch procedure, the mortality rate of TOF patients was 8.5% for 10 years. This was comparable to TOF patients as reported by Cuypers et al. during their third and fourth decade of life [17]. Altogether, 71% of our deceased ACHD patients had a cardiovascular cause of death.

As expected, hospitalization rates were significant in our ACHD patients. During the study period, 63% of individuals had been hospitalized while 86% of those hospitalizations were related to cardiocasvular reasons. Hospitalization rates per year were significantly increased in patients with CHD compared to age-matched healthy controls and were particularly high in those with severe CHD. Similarly, a 10-year observation period from 2003 to 2012, as assessed by the United States Nationwide Inpatient Sample Database, found a considerable increase (up to 81.5%) in hospitalizations of ACHD patients [18], independent of the reasons for hospitalization—treating or preventing complications and sequelae of the CHD and of comorbidities. These numbers reflect the increasing demands for medical care of an aging ACHD population, yet significantly earlier than expected from biological age [9]. Somewhat exaggeratedly expressed, it may be speculated that CHD patients exhibit progeria. For example, mortality of our middle-aged patients with severe CHD corresponded to the estimated 10-year risk for fatal cardiovascular events of the general population in men ≥ 60 years of age or in women ≥ 70 years of age exhibiting several risk factors like high blood pressure, hypercholesterinemia and smoking according to the ESC SCORE [19]. Whereas recent nationwide campaigns have focused on prevention of cardiovascular risk factors to avoid early development and late complications of cardiovascular diseases in the general population, knowledge of primary care physicians in Germany on care of ACHD patients is low [3] and ACHD patients do not often seek regular advice from ACHD specialists [20, 21]. Circumstances of pre-aging, elevated morbidity and mortality caused by cardiovascular and non-cardiovascular reasons emphasize the need for close and specialized surveillance of long-term ACHD survivors. This will improve outcome and shift survival closer to the general age-matched population, especially in patients with moderate and severe CHD.

In summary, our study showed significantly increased mortality in middle-aged ACHD patients with moderate and severe CHD over a follow-up period of 15 years. Morbidity was also impressively high, as there was a high demand for in-hospital cardiovascular interventions, particularly in patients with severe CHD. Moving forward, prevention of these events and timely intervention by ACHD specialists is of paramount importance in the care of ACHD patients.

## Summary and conclusions

It is a substantial finding that this longitudinal observation study over 15 years on a well-defined cohort of ACHD confirms the findings of other studies with retrospective analysis of multicenter registry data or single-center data on high morbidity and mortality. It was not clear at that time, when the study started in 2003, to what extent ACHD patients are compromised concerning their health and life perspective. We believe, that our data derived 15 years later are a valuable endorsement of the concept [1] that CHD is a life-long chronic condition requiring long-term specialized care.

## Limitations

The LC1 study was conducted at the Goettingen Heart Center to assess ACHD patients in an early era of the growing ACHD population, as at this time there was a lack of extensive data published on this topic. It was the aim of the LC2 study to figure out how this particular and well-described study population fared during the 15 subsequent years between LC1 and LC2. This longitudinal observational design over 15 years is in contrast to retrospective analyses of larger registries. This is the strength of the present study.

Loss of follow-up was remarkably low (13%), but may have influenced our results, as the fate of these individuals is unclear. However, more patients with mild CHD were lost to follow-up than patients with severe CHD. Many factors may have played into this, from the perceived lack of need for follow-up visits by mild CHD patients, or more often changes in their place of residence as more individuals with mild CHD could not be reached after a period of 15 years. Furthermore, 12% of patients declined to participate in LC2 (45/364). The reasons were not precisely queried during data collection. Neither regular cardiology examinations at our tertiary ACHD facility nor higher severity of the CHD was a guarantee for participation in LC2. It may be speculated that these patients lacked an interest in participating in our repeat study or that they objected to take a closer look at their heart disease. Data presented was influenced by the fact that, when compared to previous studies, fewer patients with mild CHD were included in LC1 and LC2, reflecting the care of ACHD patients with moderate and severe CHD in a large tertiary ACHD center. Taking the limitations of this study into account, it will definitely be useful to conduct studies on ACHD patients in the future as multicenter studies on longitudinal observational basis.

## Appendix

See below Tables

**Table 4:** STROBE Statement—checklist of items that should be included in reports of observational studies

	Item No	Recommendation	Page no
Title and abstract	1	(*a*) Indicate the study’s design with a commonly used term in the title or the abstract	1, 3, 7
		(*b*) Provide in the abstract an informative and balanced summary of what was done and what was found	3
Introduction			
Background/rationale	2	Explain the scientific background and rationale for the investigation being reported	6
Objectives	3	State specific objectives, including any prespecified hypotheses	6
Methods			
Study design	4	Present key elements of study design early in the paper	6
Setting	5	Describe the setting, locations, and relevant dates, including periods of recruitment, exposure, follow-up, and data collection	6, 7
Participants	6	(*a*) *Cohort study*—give the eligibility criteria, and the sources and methods of selection of participants. Describe methods of follow-up *Case–control study*—give the eligibility criteria, and the sources and methods of case ascertainment and control selection. Give the rationale for the choice of cases and controls *Cross-sectional study*—give the eligibility criteria, and the sources and methods of selection of participants	6, 7
		(*b*) *Cohort study*—for matched studies, give matching criteria and number of exposed and unexposed *Case–control study*—for matched studies, give matching criteria and the number of controls per case	%
Variables	7	Clearly define all outcomes, exposures, predictors, potential confounders, and effect modifiers. Give diagnostic criteria, if applicable	7
Data sources/ measurement	8*	For each variable of interest, give sources of data and details of methods of assessment (measurement). Describe comparability of assessment methods if there is more than one group	7
Bias	9	Describe any efforts to address potential sources of bias	%
Study size	10	Explain how the study size was arrived at	6, 7, 8
Quantitative variables	11	Explain how quantitative variables were handled in the analyses. If applicable, describe which groupings were chosen and why	7, 8
Statistical methods	12	(*a*) Describe all statistical methods, including those used to control for confounding	7, 8
		(*b*) Describe any methods used to examine subgroups and interactions	6, 7
		(*c*) Explain how missing data were addressed	%
		(*d*) *Cohort study*—if applicable, explain how loss to follow-up was addressed *Case–control study*—if applicable, explain how matching of cases and controls was addressed *Cross-sectional study*—if applicable, describe analytical methods taking account of sampling strategy	6, 7,
		(*e*) Describe any sensitivity analyses	%

4 and

**Table 5:** Results

Participants	13*	(a) Report numbers of individuals at each stage of study—e.g., numbers potentially eligible, examined for eligibility, confirmed eligible, included in the study, completing follow-up, and analyzed	8
		(b) Give reasons for non-participation at each stage	8
		(c) Consider use of a flow diagram	Fig. 1
Descriptive data	14*	(a) Give characteristics of study participants (e.g., demographic, clinical, social) and information on exposures and potential confounders	8–16
		(b) Indicate number of participants with missing data for each variable of interest	8–12
		(c) *Cohort study*—summarize follow-up time (e.g., average and total amount)	6, 8, 9
Outcome data	15*	*Cohort study*—report numbers of outcome events or summary measures over time	Figs. 3, 4, 5
		*Case–control study—*report numbers in each exposure category, or summary measures of exposure	*%*
		*Cross-sectional study—*report numbers of outcome events or summary measures	*13, 14, 15, 16*
Main results	16	(*a*) Give unadjusted estimates and, if applicable, confounder-adjusted estimates and their precision (e.g., 95% confidence interval). Make clear which confounders were adjusted for and why they were included	8, 13, 14, 15, 16
		(*b*) Report category boundaries when continuous variables were categorized	%
		(*c*) If relevant, consider translating estimates of relative risk into absolute risk for a meaningful time period	%
Other analyses	17	Report other analyses done—e.g., analyses of subgroups and interactions, and sensitivity analyses	Figs. 3, 4, 5
Discussion			
Key results	18	Summarize key results with reference to study objectives	17, 18, 19
Limitations	19	Discuss limitations of the study, taking into account sources of potential bias or imprecision. Discuss both direction and magnitude of any potential bias	20
Interpretation	20	Give a cautious overall interpretation of results considering objectives, limitations, multiplicity of analyses, results from similar studies, and other relevant evidence	17, 18, 19
Generalisability	21	Discuss the generalisability (external validity) of the study results	17, 18, 19
Other information			
Funding	22	Give the source of funding and the role of the funders for the present study and, if applicable, for the original study on which the present article is based	1, 19

^5^.

## Abbreviations

ACHD: Adults with congenital heart defect
AR: Aortic regurgitation
AS: Aortic stenosis (valvulär/subvalvulär/supravalvulär)
ASD: Atrial septal defect
CHD: Congenital heart defect
ccTGA: Congenitally corrected transposition of the great arteries
dTGA: Dextroposition transposition of the great arteries
DILV: Double inlet left ventricle
DORV: Double outlet right ventricle
EPS: Electrophysiological study
F: Female
Fig.: Figure
HR: Hazard ratio
IQR: Interquartile range
LC: Life chances
M: Male
n.s: Not significant
Py: Patient years
PA: Pulmonary atresia
SOEP: German Socio-Economic Panel
TOF: Tetralogy of Fallot
TA: Tricuspid atresia
VF: Ventricular fibrillation
VSD: Ventricular septal defect
VT: Ventricular tachycardia
